# Supplementary figures and images for: TACI regulates marginal zone B cell development
Source: J Exp Med. 2026 May 5;223(6):e20251308. doi: 10.1084/jem.20251308 (PMC13142658; doi:10.1084/jem.20251308)

Source Data Fig 6G

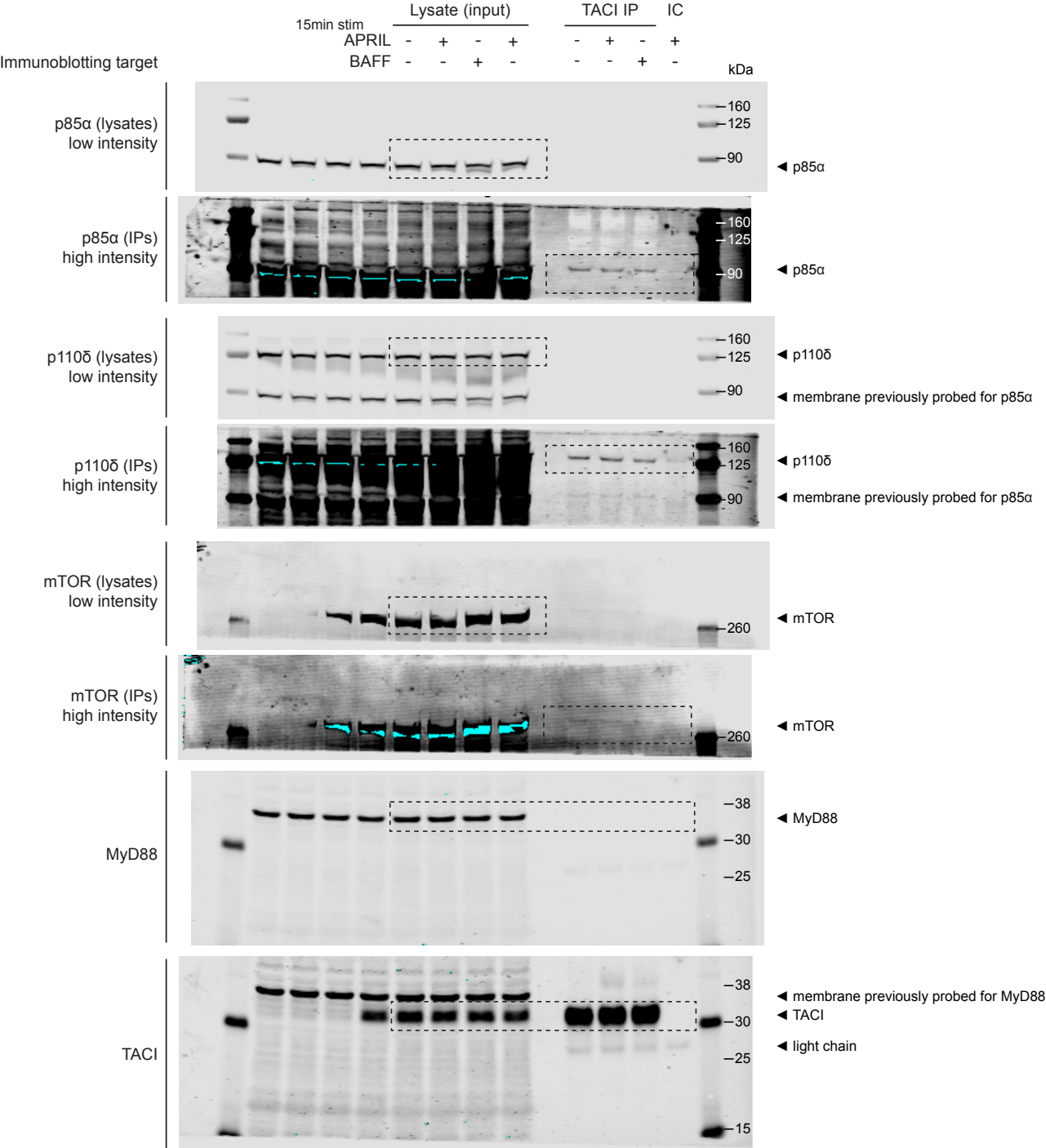

Supplement: SourceData F6 — is the source file for Fig. 6. [file jem_20251308_sourcedataf6.pdf]
